# CAGE-seq analysis of Epstein-Barr virus lytic gene transcription: 3 kinetic classes from 2 mechanisms

**DOI:** 10.1371/journal.ppat.1007114

**Published:** 2018-06-04

**Authors:** Reza Djavadian, Mitchell Hayes, Eric Johannsen

**Affiliations:** 1 Department of Medicine, University of Wisconsin School of Medicine and Public Health, Madison, Wisconsin, United States of America; 2 Department of Oncology (McArdle Laboratory for Cancer Research), University of Wisconsin School of Medicine and Public Health, Madison, Wisconsin, United States of America; Tulane Health Sciences Center, UNITED STATES

## Abstract

Epstein-Barr virus (EBV) lytic replication proceeds through an ordered cascade of gene expression that integrates lytic DNA amplification and late gene transcription. We and others previously demonstrated that 6 EBV proteins that have orthologs in β- and γ-, but not in α-herpesviruses, mediate late gene transcription in a lytic DNA replication-dependent manner. We proposed a model in which the βγ gene-encoded viral pre-initiation complex (vPIC) mediates transcription from newly replicated viral DNA. While this model explains the dependence of late gene transcription on lytic DNA replication, it does not account for this dependence in α-herpesviruses nor for recent reports that some EBV late genes are transcribed independently of vPIC. To rigorously define which transcription start sites (TSS) are dependent on viral lytic DNA replication or the βγ complex, we performed Cap Analysis of Gene Expression (CAGE)-seq on cells infected with wildtype EBV or EBV mutants defective for DNA replication, βγ function, or lacking an origin of lytic replication (*Ori*Lyt). This approach identified 16 true-late, 32 early, and 16 TSS that are active at low levels early and are further upregulated in a DNA replication-dependent manner (leaky late). Almost all late gene transcription is vPIC-dependent, with BCRF1 (vIL10), BDLF2, and BDLF3 transcripts being notable exceptions. We present evidence that leaky late transcription is not due to a distinct mechanism, but results from superimposition of the early and late transcription mechanisms at the same promoter. Our results represent the most comprehensive characterization of EBV lytic gene expression kinetics reported to date and suggest that most, but not all EBV late genes are vPIC-dependent.

## Author summary

Herpesvirus lytic replication is characterized by the expression of early genes prior to viral DNA replication followed by late gene expression. Late genes are not only expressed after DNA replication, but cannot be expressed in its absence. We and others have shown that γ-herpesviruses, such as Epstein-Barr virus (EBV), as well as β-herpesviruses accomplish late gene transcription using a viral pre-initiation complex (vPIC). Proteins in this complex are encoded by genes with orthologs found only in β- and γ-herpesviruses (βγ genes) and absent in α-herpesviruses. We used EBV mutants defective for DNA replication or βγ complex/vPIC function to rigorously identify all EBV late genes and determine the extent of their dependence upon vPIC for expression. Because of the extensive bidirectional transcription of the EBV genome during lytic replication, unambiguous quantification of most lytic transcripts is not possible by conventional methods such as RNA-seq and RT-qPCR. We therefore used deep sequencing of non-amplified 5’ ends to uniquely localize and quantify transcription start sites in a strand-specific manner. Our results demonstrate that most, but not all, EBV late transcripts require vPIC for their expression. Furthermore, our results indicate that transcription that appears to be partially dependent on DNA replication (called “leaky” late transcription) results from superimposed early and late transcriptional mechanisms acting on the same promoter.

## Introduction

Epstein-Barr virus (EBV) is a γ-herpesvirus that infects more than 95% of the human adult population. If acquired early in life, EBV infection is generally asymptomatic, however infection in adolescence may lead to infectious mononucleosis [1,2]. EBV infection is associated with a wide-spectrum of malignancies, such as Burkitt lymphoma, Hodgkin lymphoma, diffuse large B cell lymphoma, NK/T-cell lymphoma, post-transplant lymphoproliferative disease, nasopharyngeal carcinoma, and gastric carcinoma [3–6]. Although these tumors are characterized by latent EBV infection, an increasing body of evidence indicates that lytic infection is also important for the emergence of EBV-associated malignancies [7–10].

Herpesvirus lytic replication proceeds through a highly ordered cascade of gene expression that integrates viral DNA synthesis with structural protein production, while evading the host immune response. In EBV, this process is initiated by two immediate early viral transcription factors Zta (also called Z, ZEBRA, EB1—encoded by *BZLF1)* and Rta (also called R–encoded by *BRLF1)* [11–16], which activate early gene promoters leading to expression of proteins essential for viral DNA synthesis [12,17–19]. Late genes, which mainly encode structural proteins, have long been defined by their strict dependence on viral DNA replication for expression, but the basis for this dependence has been elusive. A key advance in our understanding of late gene transcription was the discovery that β- and γ-herpesviruses transcribe their late genes by a mechanism fundamentally different than the α-herpesviruses. This discovery began with the finding that several genes with orthologs found only in β- and γ-herpesviruses (βγ genes) were essential for late gene expression, but dispensable for DNA replication [20–27]. One of these βγ genes was shown to encode a viral TATA binding protein (vTBP) [28,29]. Studies of the EBV vTBP, encoded by *BcRF1*, showed that it preferentially bound to an atypical TATA box (TAT**T**) found in many late gene promoters and activated transcription through this element in reporter assays [28]. Subsequently, we and others showed that five other EBV βγ genes are essential for late gene expression and activation of TATT-containing promoters [30–32]. Although the precise roles played by each gene are yet to be defined, they have been demonstrated to form a protein complex with vTBP, termed the viral pre-initiation complex (vPIC) [31]. We demonstrated that vPIC can only mediate late gene expression when the EBV origin of replication (*Ori*Lyt) is present *in cis* and proposed a model in which EBV vPIC mediates late gene expression by recruiting RNA polymerase II to use newly replicated viral DNA as the template for transcription [30].

Although this model provides a mechanistic link between lytic DNA replication and late gene transcription in β- and γ-herpesviruses, it does not explain why this dependence is also found in α-herpesviruses. This exception also raises the question of whether and to what extent EBV late gene expression might be accomplished by vPIC-independent mechanisms. In order to answer this question, it is essential to first distinguish genes that are strictly dependent upon DNA replication (late or true late) from those that are partially dependent (leaky late) as well as from early genes which exhibit no dependence upon DNA replication for their expression. The most comprehensive attempt at such an analysis of EBV lytic gene kinetics published to date was by Yuan et al. [33], which employed a custom oligo array to detect EBV lytic transcripts expressed in Akata cells in response to B cell receptor signaling. While this study was a significant advance in our understanding of late gene expression, it had several important limitations: First, it relied on phosphonoacetic acid (PAA) to block viral DNA replication. This approach identified many late genes, but also impaired expression of many well-characterized early genes such as *BMRF1*. For genes that were less well-characterized, it was unclear the extent to which partial dependence upon DNA replication was a real phenomenon or attributable to transcript overlap or other artifacts such as PAA toxicity. The EBV DNA replication defective mutants described in our recent study lacking the single-stranded DNA binding protein BALF2, which permits *trans*-complementation of DNA replication, as well the *Ori*Lyt knockout [30], afford the opportunity to study EBV transcript dependence upon DNA replication and *Ori*Lyt directly, without the toxicity of DNA polymerase inhibitors. Using the same approach, we can assess transcript dependence on the vPIC complex by *trans*-complementing EBV mutants deleted for specific βγ genes.

A major impediment to any genome-wide analysis of the EBV lytic transcriptome is that transcripts overlap so extensively that most transcripts cannot be unambiguously quantified by any conventional high-throughput method, including RNA-seq, RT-qPCR, and oligo arrays. Recently, quantitative high throughput methods have been developed to identify and quantify transcription start sites (TSS) which can circumvent the barrier presented by extensively overlapping transcripts. We used one of these methods: **n**on-**A**mplified **n**on-**T**agging **I**llumina **C**ap **A**nalysis of **G**ene **E**xpression (nAnT-iCAGE [34], hereafter referred to as CAGE-seq) to quantify each EBV lytic transcript, its transcription start site (TSS), and its dependence on DNA replication, the presence of an *Ori*Lyt, and on the βγ gene-encoded vPIC. This approach not only allowed us to comprehensively define the kinetics of the EBV lytic transcriptome, but also provided insights into the unique features of the early and late transcriptional mechanisms.

## Results

### Overlapping nature of EBV lytic transcripts confounds quantitative analysis by conventional methods

**Fig 1:**
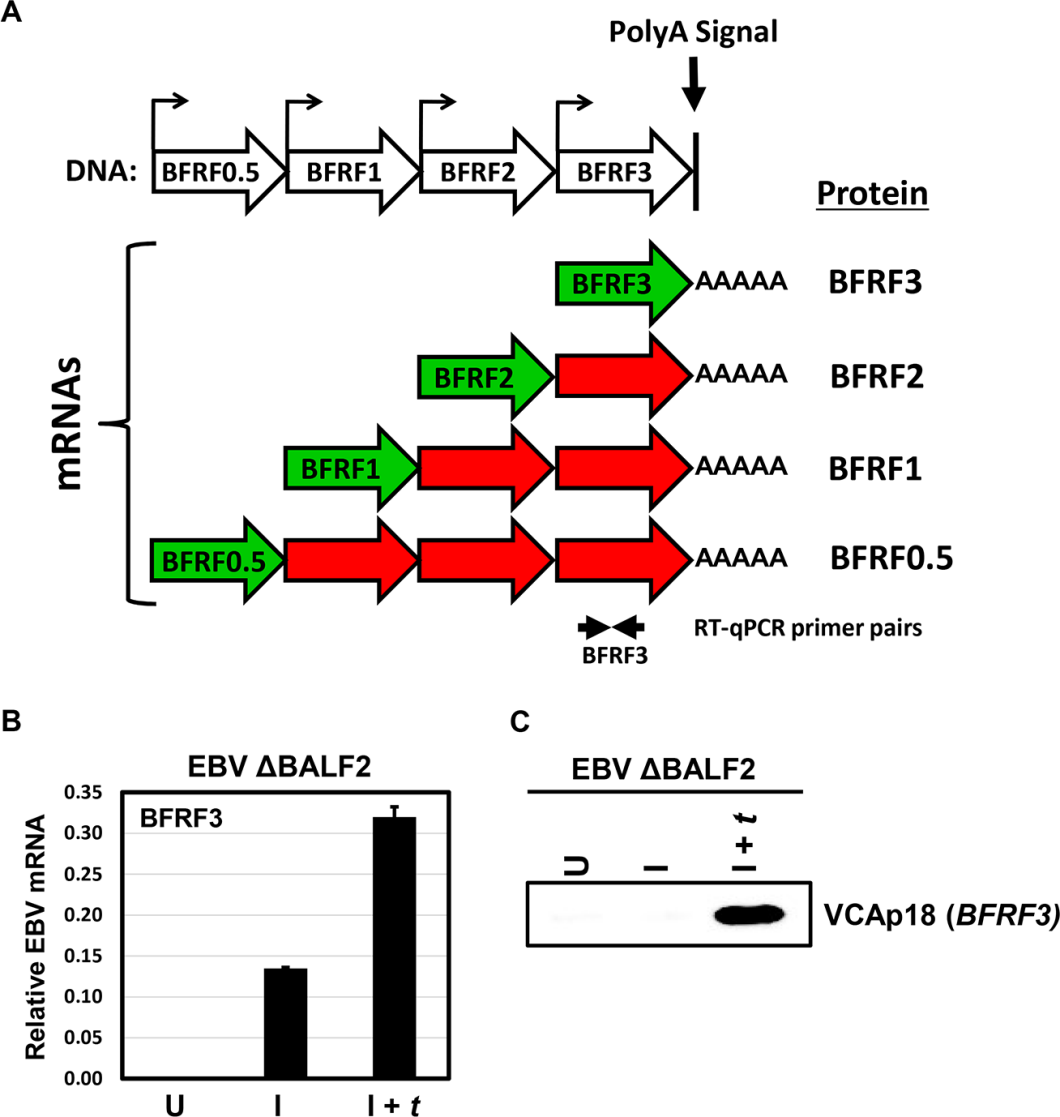
Nested structure of EBV lytic transcripts confounds quantification of EBV lytic mRNAs. A) Schematic of the BFRF0.5-BFRF1-BFRF2-BFRF3 nested lytic transcriptional unit, the overlapping structure of which is shared by many EBV lytic transcripts due to frequent use of shared polyA signal sequences. B) Bar plot showing EBV BFRF3 mRNA signal relative to cellular β-actin as measured by RT-qPCR in EBV ΔBALF2 HEK293 cells that were either uninduced (U), induced with transfection of Rta and Zta expression plasmids (I), or induced and *trans*-complemented with transfection of Rta, Zta, and BALF2 expression plasmids (I + *t*) at 48 hours post-induction. C) Western blot for *BFRF3* protein product (VCAp18) from lysates corresponding to samples in panel B.

The EBV transcriptome is organized in clusters, where multiple mRNAs are driven by unique promoters, but use the same polyA signal and are therefore co-terminal. One such cluster is the BFRF0.5-BFRF1-BFRF2-BFRF3 locus shown in Fig 1A. In the absence of an IRES, only the first ORF is efficiently translated, leading to transcripts that are overlapping, but not polycistronic. This organization of transcripts, which is typical, presents technical challenges, limiting the ability to precisely measure the mRNA levels of all, except the longest transcript, by RT-qPCR or RNA-seq. To demonstrate this phenomenon, EBV ΔBALF2 HEK293 cells, in which the gene encoding the single-stranded DNA binding protein BALF2 (required for lytic DNA replication) is disrupted (previously described in [30]]), were either uninduced (U), induced into the lytic phase by transfecting Rta and Zta expression plasmids (I) or induced and *trans*-complemented by transfecting Rta, Zta, and BALF2 expression plasmids (I + *t*) to further rescue the DNA replication defect. When RT-qPCR was employed to quantify BFRF3 mRNA levels, a signal was detected in the absence of DNA replication and increased when DNA replication was restored by BALF2 *trans*-complementation (Fig 1B, I vs I + *t*). In contrast, the *BFRF3* protein product VCAp18 was detectable by western blotting only in the presence of EBV lytic DNA replication (Fig 1C, I + *t*). This apparent discrepancy arises from the inability of RT-qPCR to distinguish authentic BFRF3 transcripts from overlapping early transcripts in this cluster which contain the BFRF3 sequence in their 3’ UTRs. Thus, the overlapping nature of the EBV lytic transcriptome can confound accurate assessment of EBV lytic mRNA levels.

### Cap Analysis of Gene Expression (CAGE)-seq uniquely quantifies each overlapping transcript

In order to uniquely measure each EBV lytic transcript, we performed CAGE-seq, a technique that sequences only the 5’ portion of each mRNA and provides highly quantitative, strand-specific reads that allow mapping of individual transcription start sites (TSS) [34]. Because CAGE-seq only sequences TSS, it avoids artifacts arising from transcript overlap. For these experiments, we used HEK293 cells infected with wildtype EBV, EBV mutants defective for DNA replication (Δ*Ori*Lyt or ΔBALF2), or vPIC (ΔBDLF4) [30]. Cells were either uninduced (U), induced by Rta and Zta (I), or induced and *trans*-complemented by transfection of a plasmid expressing the missing EBV protein (I + *t*). Cells were harvested for CAGE-seq at 48 hours post-induction. Resultant CAGE-seq tags were aligned to the human (GRCh38) and EBV genomes. Aligned reads were clustered using Paraclu [35] and each TSS cluster was quantified in tags per million reads mapped (TPM). In uninduced cells, EBV transcripts accounted for approximately 620 per one million mRNAs expressed (Table 1). This number increased to between about 13,000 and 44,000 TPM with induction of EBV replication. We estimate our induction protocol resulted in EBV replication in ~10% of cells, thus actual EBV transcript abundance may be as much as 10-fold higher in cells experiencing the EBV lytic cycle.

**Table 1:** Summary of EBV CAGE-seq tags detected in each cell line/condition.

Cell Line	Treatment	TPM
**ΔBALF2**	**U**	620
	**I**	23,461
	**I + *t***	23,720
**Δ*Ori*Lyt**	**I**	16,986
**ΔBDLF4**	**I**	28,544
	**I + *t***	44,140
**WT**	**I**	13,542

For each cell line and treatment, the total number of CAGE-seq tags mapping to the EBV genome are shown. Treatment conditions include: uninduced (U), induced by transfection of Rta and Zta (I), and induced by transfection of Rta and Zta and *trans*-complemented with the missing gene product (I + *t*).

**Fig 2:**
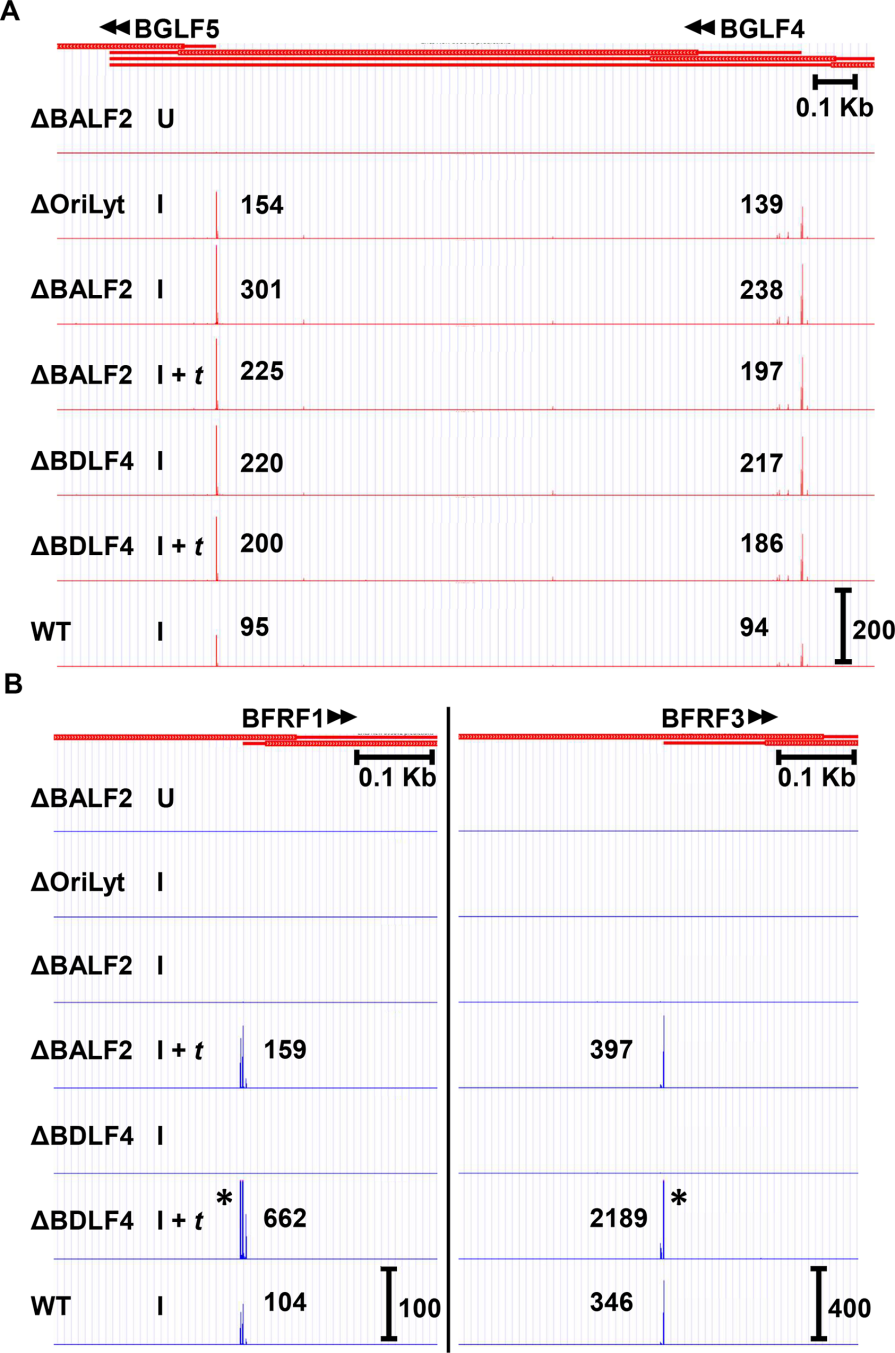
CAGE-seq readily distinguishes late from early gene transcription start sites (TSS) based on their dependence upon lytic DNA replication and the βγ-encoded vPIC. CAGE-seq tracks from EBV ΔBALF2, Δ*Ori*Lyt, ΔBDLF4, and WT infected HEK293 cells showing TSS location and transcript abundance (in tags per million (TPM)) for A) representative early genes *BGLF4* and *BGLF5* and B) representative late genes *BFRF1* and *BFRF3*. For each track, the treatment condition is indicated: cells were either uninduced (U), induced by transfection of Rta and Zta (I), or induced by transfection of Rta and Zta, and further *trans-*complemented with either BALF2 or BDLF4 (I + *t*) in the appropriate mutant cell line (ΔBALF2 or ΔBDLF4, respectively). In each case, cells were harvested for CAGE-seq 48 hours post-transfection. Scale marker in the WT track indicates track heights depicted in all tracks for the same TSS cluster. * indicates TSS signals surpassing the indicated scale. Full tracks are available to view in an interactive viewer (https://go.wisc.edu/58sxkb).

Examination of well characterized early transcription start sites such as BGLF4 and BGLF5 (Fig 2A) demonstrated that these transcripts were readily detected in the absence of DNA replication (ΔBALF2 I and Δ*Ori*Lyt I tracks) and expressed independently of the βγ gene-encoded vPIC complex (ΔBDLF4 I track). In fact, BALF2 *trans*-complementation appeared to result in a slight decrease in abundance of BGLF5 (301 vs. 225 TPM) and BGLF4 (238 vs. 197), suggesting that early and late transcription may compete for limited cellular resources. This decrease in transcription was observed for most early genes. Although it is formally possible that this observation may be due to differences in efficiency of induction of replication between different conditions, we observed similar total EBV transcripts in the ΔBALF2 I and ΔBALF2 I + *t* conditions (Table 1). Slight decreases were also observed with BDLF4 *trans*-complementation for both BGLF5 (220 vs. 200) and BGLF4 (217 vs. 186), once again suggesting that early gene transcription may compete for limiting resources with late transcription mechanisms. This decrease occurred despite the presence of more total EBV transcripts in the ΔBDLF4 I + *t* condition (44,140 TPM) compared to the ΔBDLF4 I condition (28,544 TPM), as shown in Table 1.

In contrast, transcription of canonical late genes such as *BFRF1* or *BFRF3* was not detected by CAGE-seq in the absence of DNA replication (Fig 2B, Δ*Ori*Lyt I and ΔBALF2 I tracks), but was restored with BALF2 *trans*-complementation (Fig 2B, ΔBALF2 I + *t* tracks). These genes were also fully dependent upon the βγ gene-encoded vPIC complex for their expression (Fig 2B, compare ΔBDLF4 I with ΔBDLF4 I + *t*). It is important to note that the levels and kinetics of expression of the BFRF3 transcripts detected by CAGE-seq, most closely resemble the expression patterns detected by western blotting for the *BFRF3* protein product VCAp18 (Fig 1C), in contrast to results obtained by RT-qPCR (Fig 1B). Collectively, these results indicate that CAGE-seq is a reliable method for detection of EBV transcripts on a genome-wide level and is not susceptible to artifacts arising from transcript overlap.

### Identification of non-canonical EBV late genes

**Fig 3:**
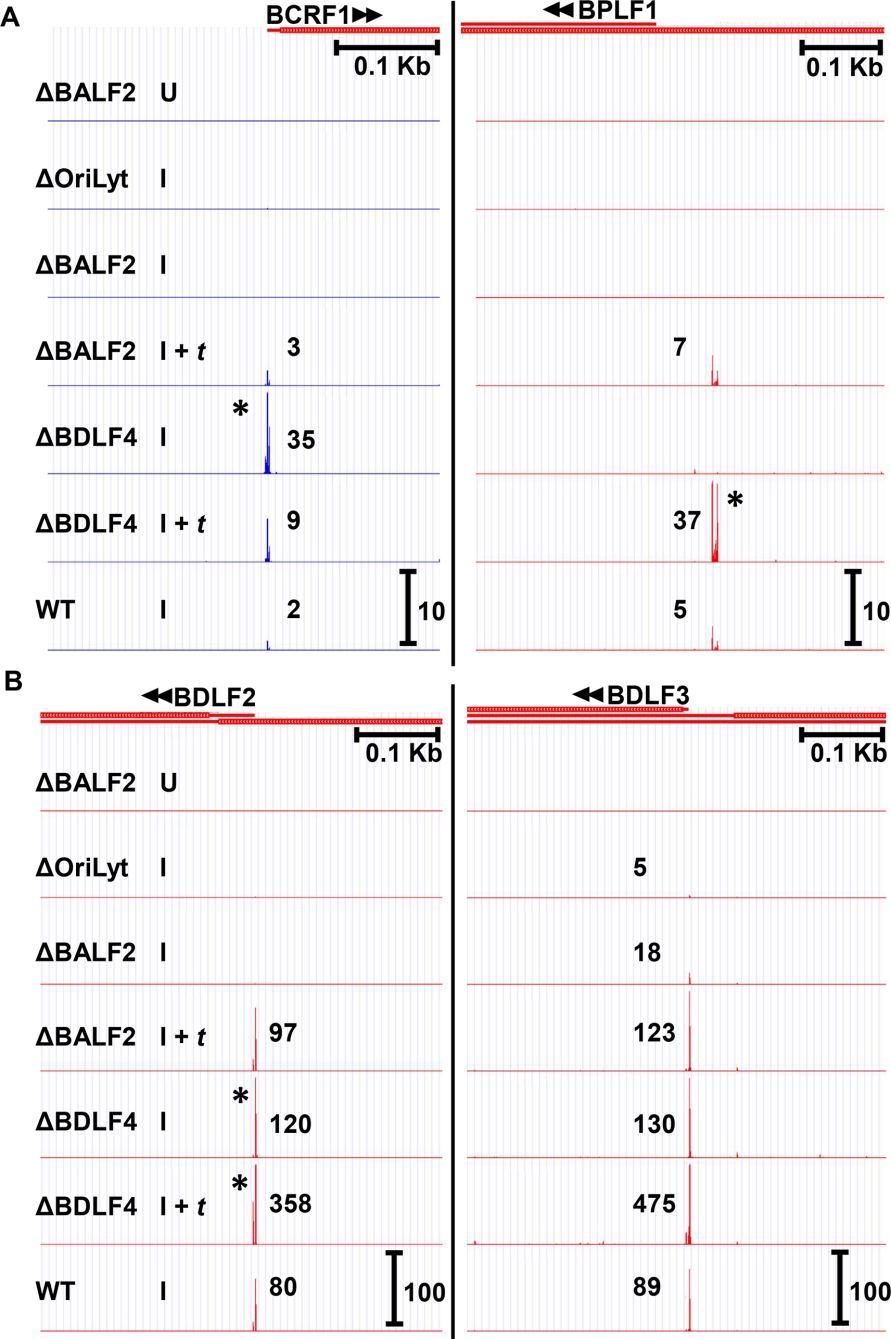
A minority of EBV late transcripts are expressed independently of the βγ-encoded vPIC. A) CAGE-seq tracks for two EBV late genes, *BCRF1* (vIL10) and *BPLF1* previously reported to be expressed independently of the βγ-encoded vPIC. B) CAGE-seq tracks for *BDLF2* and *BDLF3* showing that *BDLF2* is a late gene expressed independently of the βγ-encoded vPIC, whereas *BDLF3* exhibits partial dependence upon DNA replication (i.e., “leaky” late kinetics). BDLF2 and BDLF3 transcripts are strongly detected in the absence of BDLF4 (ΔBDLF4 I), but increase upon BDLF4 *trans*-complementation (ΔBDLF4 (I + *t*)). Treatment conditions are similar as described for Fig 2. Scale marker in the WT track indicates track heights depicted in all tracks for the same TSS cluster. * indicates TSS signals surpassing the indicated scale. Full tracks are available to view in an interactive viewer (https://go.wisc.edu/58sxkb).

Although the EBV βγ gene-encoded vPIC complex requires DNA replication to mediate late gene expression, a recent report by McKenzie et al. found that at least two EBV late genes, which by definition require DNA replication, are detectable when BGLF3, an essential vPIC component is knocked down by siRNA [36]. Both of these genes, *BCRF1* (vIL-10) and *BPLF1* (the largest tegument protein), were found to be expressed at low levels (<40 TPM in all conditions, see Fig 3A) and, consistent with late kinetics, were not detectable in the absence of DNA replication (Fig 3A, Δ*Ori*Lyt I and ΔBALF2 I tracks). Our CAGE-seq data suggests that only *BCRF1* (vIL10) can be expressed in the absence of vPIC, whereas *BPLF1* is a canonical late gene that requires BDLF4 for its expression (Fig 3A, compare ΔBDLF4 I vs ΔBDLF4 I + *t* tracks). We also identified an additional candidate, non-canonical, late gene: *BDLF2* (Fig 3B). *BDLF2* exhibits strictly late kinetics and was not detectable in the absence of *Ori*Lyt or BALF2 (Δ*Ori*Lyt I or ΔBALF2 I tracks, respectively), but was expressed in the absence of BDLF4 (ΔBDLF4 I track). The BDLF2 TSS signal did, however, further increase upon BDLF4 *trans*-complementation (ΔBDLF4 I + *t* track) from 120 to 358 TPM, potentially a reflection of higher total EBV lytic transcription in this condition (Table 1). Other genes may be partially transcribed by non-canonical mechanisms. For example low level transcription of *BDLF3* was observed in the absence of *Ori*Lyt or BALF2 (Fig 3B. Δ*Ori*Lyt I and ΔBALF2 I tracks), but its TSS signal increased from 18 to 123 TPM upon BALF2 *trans*-complementation (Fig 3B. ΔBALF2 I and ΔBALF2 I + *t* tracks), consistent with leaky late kinetics. A much higher level of transcription was maintained in the absence of BDLF4 (130 TPM, Fig 3B. ΔBDLF4 I track), suggesting that this leaky late expression may be non-canonical. In summary, our CAGE-seq data confirm that some late genes can be expressed independently of vPIC. These include *BCRF1* (vIL10), *BDLF2*, and likely the leaky late gene *BDLF3*. In contrast, our results suggest that *BPLF1* is a canonical late gene, fully dependent on vPIC for its expression.

### Genome-wide classification of EBV lytic genes based on their dependence on DNA replication and the βγ-encoded vPIC complex for expression

In an effort to systematically organize EBV lytic genes according to their kinetic class, we calculated the ratio of the CAGE-seq signal (from S1 Table) in ΔBALF2 I condition to that observed in the *trans-*complemented ΔBALF2 I + *t* condition. As expected, well-established late genes had BALF2 ratios near 0 and early genes had much higher ratios, often greater than 1. As previously stated, it is likely that this increase in early gene transcription in the absence of DNA replication is due to the competition between late gene and early gene transcription. We tentatively classified all genes with BALF2 ratios less than 0.1 as true late genes, those above 0.5 as early, and those with intermediate ratios as “leaky” late genes (Table 2). Based on these criteria, EBV replication is characterized by expression of at least 32 genes with early kinetics, 16 with late, and 16 genes that are expressed with leaky late kinetics. We only identified one TSS that exhibited latent kinetics, the C promoter; however, we did observe some early TSS in the uninduced state, consistent with low level spontaneous replication. As has been reported in other genome-wide analysis of EBV replication [37,38], we observed a large number of TSS that did not correspond to annotated transcripts. While many of these were transcribed at low levels, some “unknown” TSS clusters were expressed at much higher levels (S1 Table). In one case (BFRF0.5), a strong TSS was present just 3’ to the annotated open reading frame, suggesting that the authentic protein product might be initiated from the internal methionine at annotated codon 51. Because this TSS is almost certainly responsible for the early signal observed with the BFRF3 primers (Fig 1B) and because the BFRF0.5 initiator methionine has not be empirically determined, we included this TSS as a possible BFRF0.5 transcript in Table 2. Indeed, during the revision of this manuscript, Bencun et al. published ribosomal profiling experiments that also implicate codon 51 as a likely alternative start site for BFRF0.5 (called BFRF1a in their study) [39]. A similar issue arose with a cluster just upstream of BNLF2a that may result in a 5’ UTR that is too short to allow translation of BNLF2a, but could encode BNLF2b. This TSS was labeled BNLF2a/b to reflect this ambiguity.

**Table 2:** EBV lytic transcription start sites (TSS) detected by CAGE-seq and their dependence upon DNA replication (BALF2 Ratio) and the βγ gene-encoded vPIC (BDLF4 Ratio).

Cluster			Strand	Kinetics	BALF2Ratio	BDLF4Ratio	WT TPM	ORF/ promoter	Annotation
Start	End								
1709	1729		+	late	0.00	0.01	25	BNRF1	major tegument protein
11330	11349		+	latent	1.02	0.81	184	Cp	latency promoter
53782	53794		+	early	0.81	1.25	758	BHRF1	v-Bcl2
58113	58119		+	late	0.00	0.00	22	BFRF0.5	terminase subunit
58581	58593		+	early	0.98	1.18	151	BFRF0.5?	terminase subunit
58860	58867		+	late	0.01	0.00	104	BFRF1	capsid nuclear egress
61372	61381		+	late	0.02	0.00	346	BFRF3	capsid—hexon tip
62231	62239		+	early	1.18	0.88	78	Fp	
75047	75054		+	leaky	0.19	0.05	72	BORF1	capsid - 1x triplex
76198	76213		+	early	1.05	1.30	161	BORF2	RNR—large subunit
78831	78840		+	early	0.93	1.26	279	BaRF1	RNR—small subunit
79869	79874		+	early	1.29	1.31	171	BMRF1	processivity factor, DNA polymerase
80811	80863		+	early	0.64	0.84	158	BMRF2	virion glycoprotein
86914	86917		+	early	0.57	0.24	30	BSRF1	virion protein, palmytoylated
88540	88546		+	leaky	0.36	0.27	66	BLRF1	glycoprotein N
88894	88898		+	leaky	0.10	0.04	366	BLRF2	tegument protein
105040	105049		+	early	0.98	1.07	200	BRRF1	Na
106271	106278		+	leaky	0.28	0.34	42	BRRF2	tegument protein
109934	109941		+	leaky	0.35	0.57	22	BKRF2	glycoprotein L
110175	110180		+	early	1.24	1.04	300	BKRF3	uracil DNA glycosylase
110924	110929		+	early	0.95	1.18	167	BKRF4	tegument protein
113906	113915		+	leaky	0.24	0.14	11	BBRF1	portal
115794	115797		+	leaky	0.47	0.40	6	BBRF2	
119129	119134		+	leaky	0.23	0.29	93	BBRF3	glycoprotein M
137247	137251		+	leaky	0.23	2.20	12	BcRF1	vTBP; vPIC component
144610	144618		+	late	0.01	0.00	5	BXRF1	
145333	145340		+	early	0.98	0.63	9	BVRF1	portal "cork"
147752	147758		+	late	0.02	0.01	16	BVRF2	scaffold protease
148650	148655		+	leaky	0.27	0.26	52	BdRF1	capsid scaffold protein
165497	165499		+	early	1.29	1.06	186	BARF1	CSF-1 decoy receptor
167605	167603		-	early	0.64	2.41	6	BNLF2a	TAP inhibitor
167499	167485		-	early	0.66	0.21	198	BNLF2a/b	TAP inhibitor/unknown
165414	165410		-	early	1.94	0.87	127	BALF1	putative vBcl-2
164786	164778		-	early	1.61	0.86	44	BALF2	ssDNA binding protein
161637	161632		-	leaky	0.46	0.98	9	BALF3	terminase subunit, pac binding
159340	159332		-	leaky	0.34	0.14	50	BALF4	glycoprotein B
150544	150530		-	late	0.00	0.00	197	BILF2	vGPCR
148156	148151		-	early	1.08	1.41	16	BVLF1	vPIC component
145119	145100		-	early	0.90	1.36	196	BXLF1	thymidine kinase
143282	143257		-	leaky	0.13	0.11	20	BXLF2	glycoprotein H
137683	137669		-	late	0.00	0.02	70	BcLF1	capsid protein, major
133323	133316		-	late	0.00	0.00	55	BDLF1	capsid protein, 2x triplex
132448	132441		-	late	0.01	0.33	80	BDLF2	virion glycoprotein
131078	131070		-	leaky	0.15	0.27	89	BDLF3	gp150
129350	129343		-	early	1.29	1.02	38	BDLF3.5	vPIC component
128404	128398		-	late	0.04	0.05	3	BGLF1	
126902	126893		-	late	0.03	0.01	59	BGLF2	virion protein
125140	125083		-	early	0.72	1.00	19	BGLF3	vPIC component
124087	124083		-	early	0.73	0.66	34	BGLF3.5	vPIC component
123871	123809		-	early	1.21	1.17	94	BGLF4	protein kinase
122429	122417		-	early	1.34	1.10	95	BGLF5	alkaline exonuclease
121303	121121		-	leaky	0.50	0.37	111	BBLF1	virion protein, myristoylated
119044	119040		-	early	1.25	1.08	203	BBLF2/3	helicase-primase, acc protein
114445	114361		-	early	1.05	1.15	74	BBLF4	helicase
106186	106182		-	early	1.07	1.96	5	BRLF1	Rta
102129	102126		-	late	0.02	0.00	210	BZLF2	gp42
92163	92158		-	late	0.00	0.01	171	BLLF1	gp350
90027	90019		-	early	1.11	1.40	133	BLLF2	
88489	88480		-	early	1.07	1.16	229	BLLF3	dUTPase
87030	87023		-	early	1.07	1.16	14	BSLF1	primase
84330	84323		-	early	1.05	1.08	969	SM	lytic RNA export
75294	75281		-	late	0.01	0.01	6	BOLF1	tegument protein, binds BPLF1
72163	72156		-	late	0.00	0.01	5	BPLF1	tegument protein, largest
58539	58534		-	leaky	0.19	0.14	6	BFLF1	packaging protein
57130	57048		-	early	0.79	0.41	187	BFLF2	capsid nuclear egress
52821	52315		-	early	0.51	1.80	65	BHLF1	OriLyt transcript

Shown are EBV lytic transcripts identified by CAGE-seq. For each transcription start site (TSS) cluster, the table specifies: the location (B95-8, V01555), strand (+ or -), kinetic class based on BALF2 ratio, BALF2 ratio (ratio of signal in I / I + *t* for ΔBALF2), BDLF4 ratio (ratio of signal in I / I + *t* for ΔBDLF4), transcript abundance in tags per million in wildtype EBV induced for replication (WT TPM), and the gene or promoter corresponding to the observed CAGE-seq TSS cluster. Annotation sources include [40,41].

**Fig 4:**
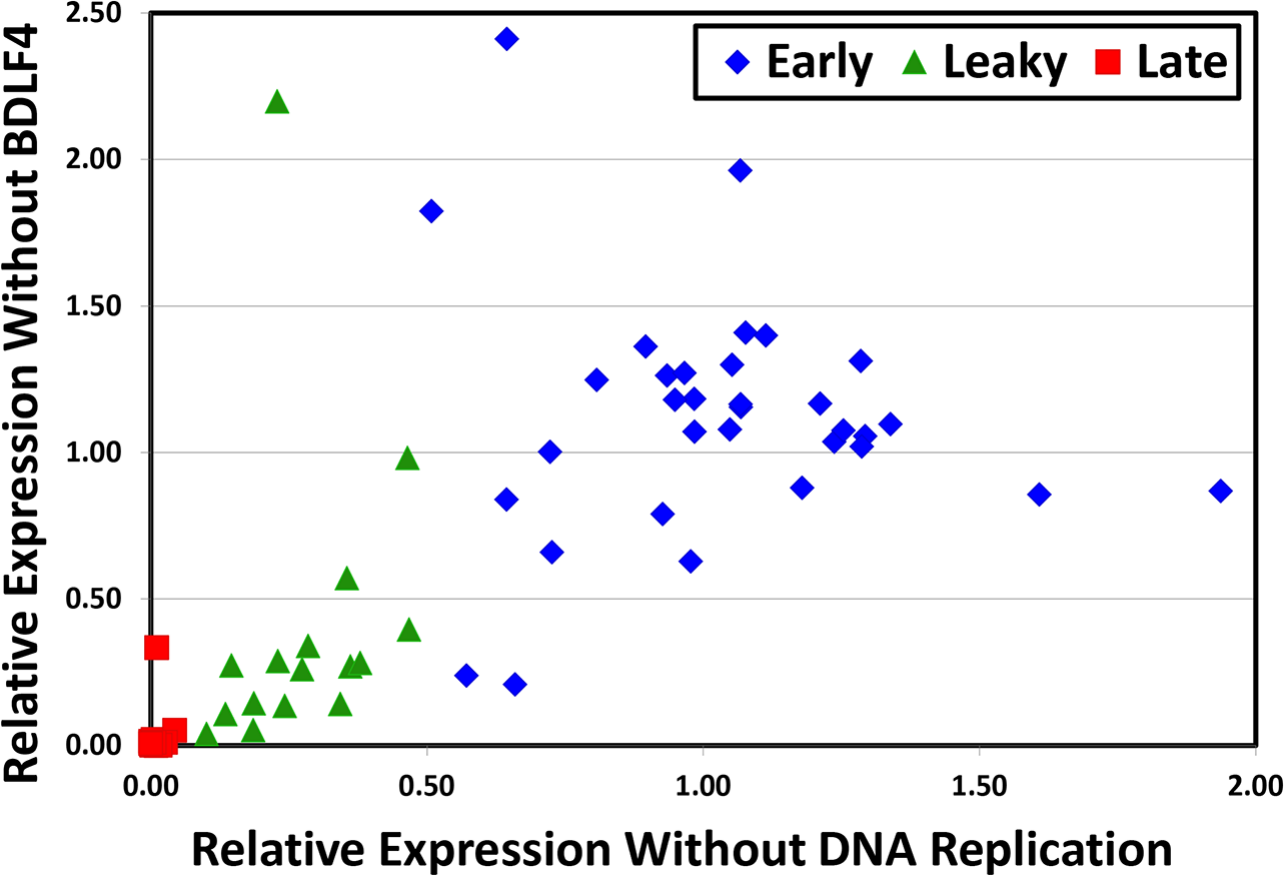
EBV transcript dependence on the βγ-encoded vPIC is highly correlated with dependence on DNA replication. Scatter plot of EBV transcripts based on their BALF2 dependence vs. BDLF4 dependence ratios determined by CAGE-seq (from Table 1). Transcripts were classified into 3 groups (early, leaky late, and late) based on their DNA replication dependence (BALF2 ratios).

We used a similar approach to estimate the dependence of each transcript on vPIC, by calculating a BDLF4 ratio, corresponding to the CAGE-seq signal in the ΔBDLF4 I condition divided by that observed in the *trans*-complemented ΔBDLF4 I + *t* condition (Table 2). Of the 16 true late genes identified here, only *BDLF2* had an elevated BDLF4 ratio (0.33), consistent with the hypothesis that the vast majority of genes with late kinetics are dependent on vPIC for their expression. *BCRF1* (vIL10) expression levels were too low to be captured by our systematic analysis (see next paragraph). Based on these BDLF4 ratios, we identified two more leaky late genes, *BcRF1* and *BALF3* that appeared to be less dependent upon vPIC than upon DNA replication. To further evaluate the extent to which dependence upon DNA replication also reflects a dependence upon vPIC for expression, we plotted the BALF2 versus the BDLF4 ratios for each kinetic class of genes (Fig 4). This confirmed that the vast majority of late genes require vPIC for expression and further demonstrated that genes exhibiting leaky late kinetics exhibit a partial dependence on vPIC proportional to their partial dependence on DNA replication.

A small number of well-annotated EBV genes were not detected as significant TSS clusters by our pipeline (details of criteria provided in the materials and methods section) and therefore are not present in Table 2. For completeness, we attempted to manually identify TSS signals in the CAGE-seq data corresponding to their known transcription start sites. This effort is presented separately in Table 3.

**Table 3:** EBV lytic transcripts not meeting high confidence CAGE-seq analysis criteria.

Cluster		Strand	Kinetics	BALF2Ratio	BDLF4Ratio	WT TPM	ORF/ promoter	Annotation
Start	End							
9660	9665	+	Late	0.03	3.94	2	BCRF1	vIL10
59502	59508	+	Early	0.96	1.74	3	BFRF2	vPIC component
124855	124862	+	Early	0.85	1.08	3	BGRF1-BDRF1	ATPase subunit of terminase
139541	139546	+	Early	1.17	1.28	3	BTRF1	
169512	169520	-	Latent	0.73	2.88	3	LMP1	LMP1
156873	156876	-	Early	2.01	0.98	2	BALF5	DNA polymerase
128979	128990	-	Early	1.18	0.08	1	BDLF4	vPIC component

Shown are CAGE-seq data for well-annotated transcripts that did not meet detection criteria (see materials and methods). Table format is the same as described for Table 2. Note that for the *BDLF4* gene, the BDLF4 ratio (I / I + *t*) may be artifactually low due to detection of the transfected BDLF4 TSS signal in the I + *t* condition.

### *BLRF2*, which encodes VCAp23, is a leaky late gene

**Fig 5:**
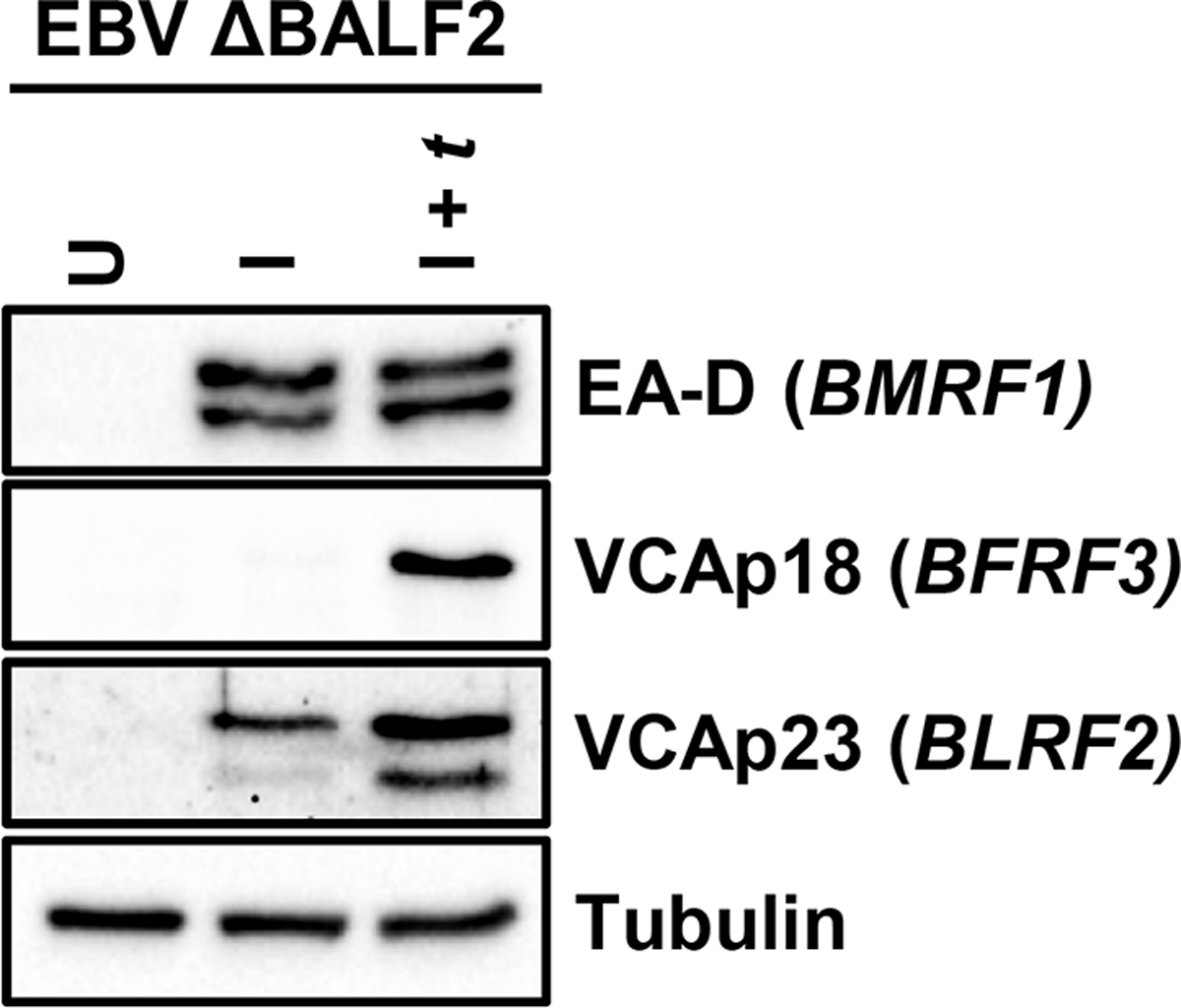
EBV *BLRF2* exhibits bona fide leaky late kinetics. Western blot for VCAp23 (product of EBV *BLRF2*) in ΔBALF2 HEK293 cells, indicating that in absence of DNA replication (induced only (I)), VCAp23 is still expressed. When DNA replication is restored by *trans*-complementation with BALF2 (I + *t*), VCAp23 levels further increase. VCAp18 (product of true late *BFRF3* gene), EA-D (product of early *BMRF1* gene) and Tubulin blots are shown as controls.

Although *BLRF2* is generally categorized as a true late gene [42–44], we detected its TSS signal in the absence of BALF2, *Ori*Lyt, and BDLF4, albeit at reduced levels. These signals dramatically increased upon BALF2 or BDLF4 *trans*-complementation, suggesting that *BLRF2* may in fact be a leaky late gene (S1 Table). Because we calculated a BALF2 ratio of 0.1, which was at the cutoff between leaky late and late genes, we chose to further investigate its kinetics. Because it is not possible to uniquely measure BLRF2 transcript levels by RT-qPCR due to overlap with the BLRF1 transcript, we assessed the *BLRF2* protein product VCAp23 by western blotting. As shown in Fig 5, in ΔBALF2 cells induced for replication (I), low level VCAp23 expression is observed and this level dramatically increases upon BALF2 *trans*-complementation (I + *t*).

### TATTWAA is the only element enriched in late gene promoters

**Fig 6:**
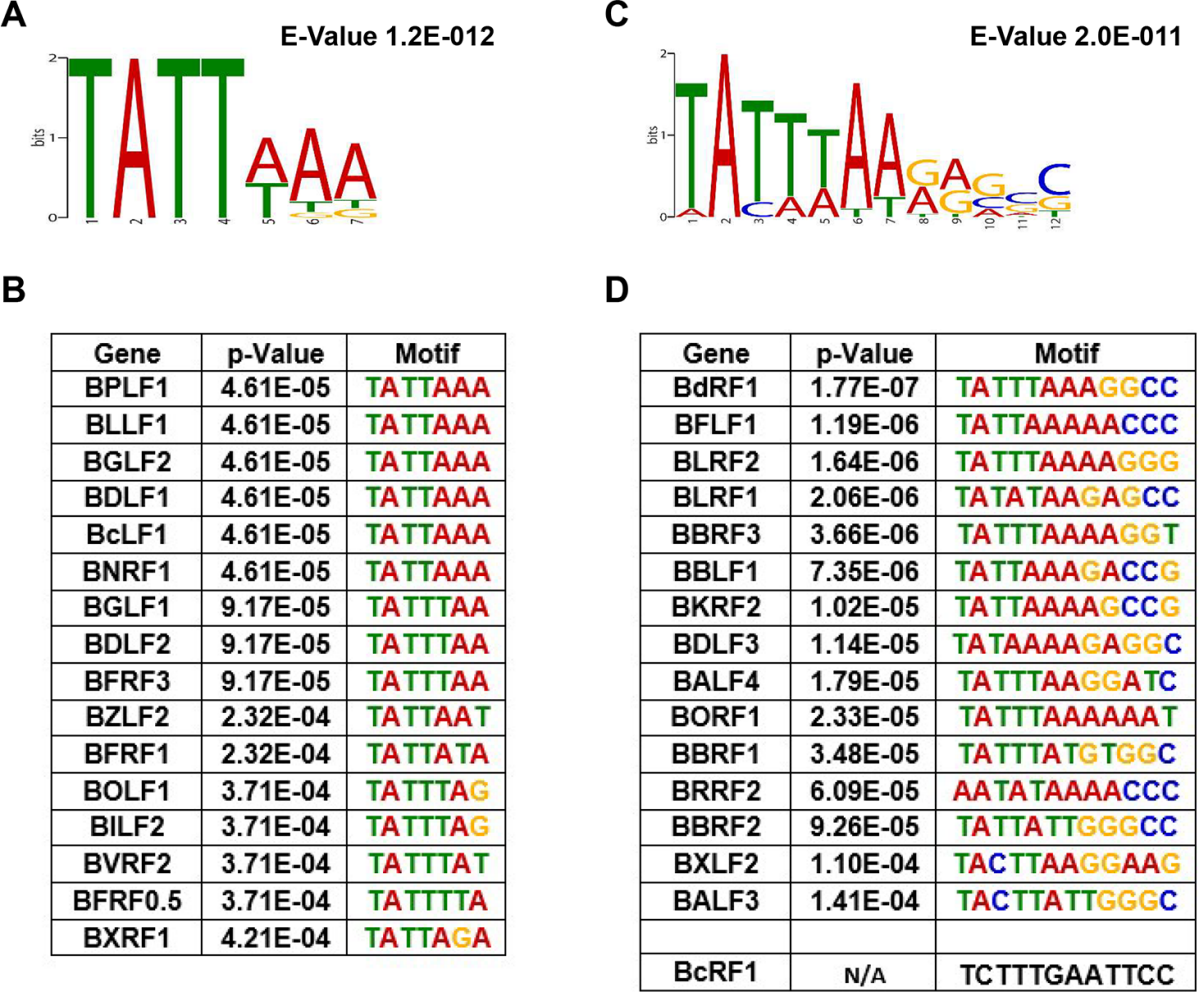
MEME analysis identifies a TATTWAA element in late and leaky late promoters. A) Consensus motif identified by MEME analysis for 16 late and C) l6 leaky late promoters from clusters in Table 1. Promoter sequences of each gene is further listed for the B) late and D) leaky late classes sorted based on conformity to the identified consensus motif. BcRF1 (vTBP) promoter sequence was excluded by MEME software due to high deviation from the determined consensus motif and is presented separately.

In an effort to determine what promoter elements determine the kinetic class of late and leaky late promoters, we performed Multiple EM for Motif Elicitation analysis (MEME) [45] of elements upstream of observed TSS with these kinetics. For both late (Fig 6A and 6B) and leaky late (Fig 6C and 6D) promoters, we found that the TATT element was enriched, but found no other sequences that were significantly enriched. The sequence was consistently found ~30bp upstream (S1 Fig) of late and most leaky late TSS and conformed to a 7 bp consensus sequence TATTWAA (where W is either A or T). There was no obvious correlation between the strength of late TSS signal and agreement with this sequence. For example, TATTTAA was present upstream of *BFRF3* and *BGLF1* (Fig 6B) which were transcribed at levels of 346 and 3 TPM (Table 2), respectively. Whereas *BILF2* and *BOLF1* had the sequence TATTTAG (Fig 6B) and were found to be transcribed at 197 and 6 TPM, respectively (Table 2). There appeared to be more deviation from this consensus in the leaky late genes with some genes such as *BRRF2* (TATAAAA) having sequences conforming to the human TBP consensus sequence [46]. TATA boxes are also observed upstream of *BDLF3* (Fig 6D) and *BCRF1* (TATAAAT) [36], which may be important for their vPIC-independent transcription. It should be noted however, that *BDLF2*, which is also transcribed independently of vPIC has the sequence (TATTTAA) to which preferential binding of BcRF1 (vTBP) would be expected.

### Leaky late kinetics can result from superimposition of early and late transcription mechanisms

**Fig 7:**
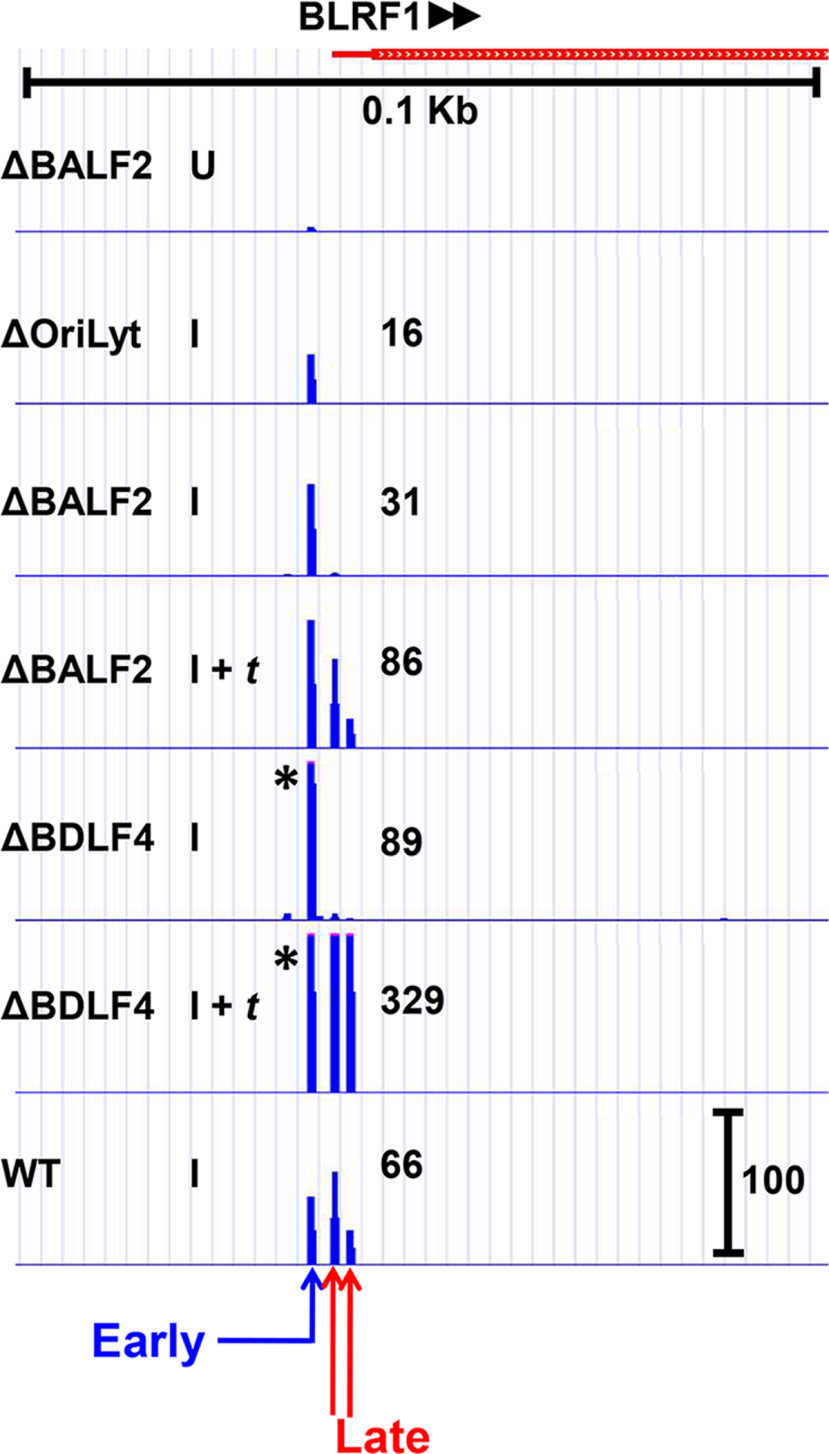
Distinct BLRF1 transcription start sites (TSS) exhibit different kinetics. CAGE-seq tracks for the BLRF1 transcript demonstrate that transcription starts in at least 3 unique locations. One TSS is independent of DNA replication and the βγ-encoded vPIC (EARLY), whereas the other two are dependent on both DNA replication and vPIC (LATE). Treatment conditions are similar as described for Fig 2. Scale marker in the WT track indicates track heights depicted in all tracks for the same TSS cluster. * indicates TSS signals surpassing the indicated scale. Full tracks are available to view in an interactive viewer (https://go.wisc.edu/58sxkb).

**Fig 8:**
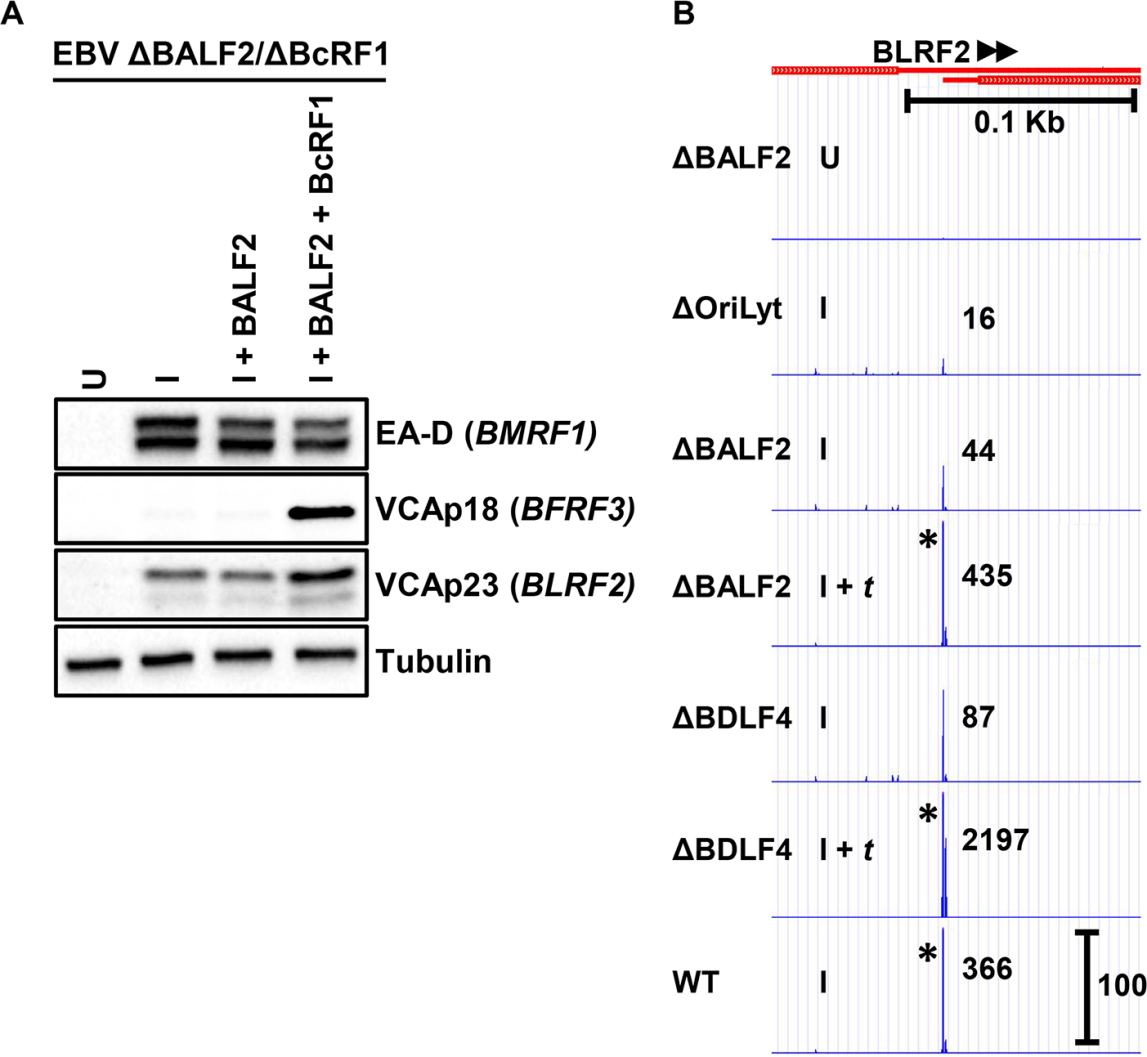
*BLRF2’s* partial dependence on DNA replication requires vPIC. A) Western blot showing levels of the *BLRF2* product VCAp23 in EBV ΔBALF2/ΔBcRF1 HEK293 cells. Cells were either uninduced (U), induced by transfection of Rta and Zta expression plasmids (I), induced by transfection of Rta and Zta expression plasmids and *trans-*complemented by transfection of either only BALF2 (I + BALF2) or both BALF2 and the essential vPIC component BcRF1 (I + BALF2 + BcRF1). B) CAGE-seq tracks corresponding to BLRF2 TSS levels. Treatment conditions are described in Fig 2. Scale marker in the WT track indicates track heights depicted in all tracks for the same TSS cluster. * indicates TSS signal surpassing the indicated scale. Full tracks are available to view in an interactive viewer (https://go.wisc.edu/58sxkb).

For most leaky late TSS clusters, we observed a uniform decrease at each position within the cluster when either lytic DNA replication (Δ*Ori*Lyt I and ΔBALF2 I) or vPIC (ΔBDLF4 I) functions were affected. However, close examination of the leaky late BLRF1 TSS cluster (Fig 7) revealed the presence of distinct TSS signals within the cluster that exhibited either early (present in all induced/I tracks) or late (absent in Δ*Ori*Lyt I, ΔBALF2 I and ΔBDLF4 I tracks) kinetics. This finding suggests that, at least in the case of *BLRF1*, the leaky late kinetics result from the production of transcripts initiating at slightly different start sites, but which ultimately encode the same open reading frame. This further suggested, given the apparent importance of vPIC for leaky late kinetics (Fig 4), that in the absence of vPIC activity, leaky late genes would revert to early kinetics. To test this hypothesis, we constructed a double mutant EBV genome in which both the single-stranded DNA binding protein BALF2 and the essential vPIC component BcRF1 (vTBP) are knocked out (i.e. ΔBALF2/ΔBcRF1) and established stable HEK293 cells. Using ΔBALF2/ΔBcRF1 HEK293 cells, we found when induced in absence of lytic DNA replication and a functional vPIC (Fig 8A, I condition) the leaky late VCAp23 protein (product of *BLRF2-*leaky late expression shown in Fig 8B*)* was expressed under early conditions. In presence of induction and *trans-*complementation with BALF2 (DNA replication restored) but in absence of BcRF1 (Fig 8A, I *+* BALF2 condition), levels of VCAp23 were not further increased. However, expressed levels of VCAp23 were increased when DNA replication was restored in presence of a functional vPIC (Fig 8A, I + BALF2 + BcRF1 condition), consistent with the hypothesis that the lytic DNA replication dependence of leaky late genes is indeed due to superimposition of canonical late transcription on a basal level of early transcription.

## Discussion

In this study, we present a comprehensive analysis of the kinetics of EBV lytic gene transcription based on dependence on lytic DNA replication and expression of *BDLF4* (a βγ gene encoding an essential component of the viral late gene pre-initiation complex (vPIC) [30,31]). We chose CAGE-seq to accurately quantify transcription start sites in a strand-specific manner. By using this approach, we were able to avoid measurement of overlapping transcripts which confounds most other genome-wide attempts to analyze EBV lytic gene expression. CAGE-seq identified 32 TSS clusters that were expressed independently of lytic DNA replication (i.e. early kinetics) and 16 TSS that exhibited late kinetics defined by their strict dependence upon lytic DNA replication. In addition, 16 TSS were identified that exhibited partial dependence on lytic DNA replication (referred to here as leaky late, but also termed early-late or γ1). Although BALF2 has been suggested to play a role in recruiting Zta to the BHLF1 promoter [47], we consistently found that genes requiring BALF2 for their expression also required *Ori*Lyt (S1 Table). Thus, for each TSS, BALF2-dependence reflects lytic DNA replication dependence.

Based on this measure, we propose reclassification (see reference [33]) of 13 genes previously identified as late (*BORF1*, *BLRF1*, *BLRF2*, *BRRF2*, *BKRF2*, *BBRF1*, *BBRF2*, *BBRF3*, *BdRF1*, *BALF4*, *BXLF2*, *BDLF3*, *BBLF1*) to leaky late and 4 putative late genes (*BMRF2*, *BSRF1*, *BKRF4*, and *BVRF1*) as early. We also suggest reclassification of 3 early genes (*BcRF1*, *BALF3*, and *BFLF1*) as leaky late and *BFRF1* from early to late. It bears mentioning that the calculated BALF2 ratios form a nearly continuous spectrum; our subdivision into three kinetic classes (early, leaky late, and late) is necessarily reductive and the BALF2 ratios themselves represent the most accurate description of a given gene’s kinetics. As anticipated, the majority of late genes encode structural proteins, including 3 capsid, 3 tegument, and 3 virion glycoproteins. This was also true for leaky late genes which encode 3 capsid, 3 tegument, and 6 virion glycoproteins. It is interesting to note that *BcRF1*, which encodes a vPIC component exhibited leaky late kinetics; however it did not depend upon vPIC for its expression based on its BDLF4 ratio.

In an effort to evaluate important TSS not captured by our bioinformatics pipeline, we manually examined the dependence of well-annotated transcripts. Including the results reported in Table 3, in total, we identified 37 early, 17 late, and 16 leaky late TSS. BILF1 was the only transcript for which we were unable to identify a corresponding TSS signal via CAGE-seq. Consistent with prior reports, we found a large number of unannotated TSS [37,38]. We chose to focus our analysis on well-annotated protein-coding transcripts and assigned TSS to specific genes based on prior annotated start sites or, when necessary, proximity to the ORF. However, it is possible that some “unknown” TSS represent additional transcripts for annotated ORFs with long 5’ UTRs. We also do not exclude the possibility that additional transcripts initiating at TSS reported in S1 Table will prove to have biologically significant roles in the EBV life cycle.

Our results revealed that the majority of late genes require BDLF4 for their expression and are thus vPIC-dependent, canonical late genes. This phenomenon extends to most leaky late genes which were found to exhibit a dependence on BDLF4 proportional to their dependence on BALF2 (Fig 4). We found that two late genes, *BCRF1* (vIL10) and *BDLF2*, while dependent on lytic DNA replication are vPIC-independent, and thus non-canonical late genes. Furthermore, the leaky late genes *BDLF3*, *BcRF1*, *and BALF3* appear to be less dependent upon vPIC, suggesting non-canonical late mechanisms may also contribute to their transcription. Previously, using a BGLF3 siRNA knockdown approach, *BCRF1* (vIL10) was identified as a non-canonical late gene [36]. Other candidates identified by this approach (*BPLF1*, *BSRF1*, or *BTRF1*) [36] could not be confirmed in our study. Based on calculated BALF2 ratios, our data suggests that *BSRF1* and *BTRF1* should be classified as early genes. In contrast, because *BPLF1* exhibits strict dependence on lytic DNA replication and vPIC, we classified it as a canonical late gene. It is important to note that except for *BSRF1*, we found that all of these genes were expressed at low levels during EBV replication which are likely to magnify the effect of any errors in measurement. Despite our attempts to minimize detection errors due to transcript overlap by using CAGE-seq as well as using highly specific mutant BACmids instead of siRNA knockdowns, independent confirmation of the kinetics of these genes by other investigators will be important given the existing apparent discrepancies. Nevertheless, we agree that the existence of non-canonical late genes represents an important exception to the existing model. Thus, deciphering the vPIC-independent mechanism linking late gene expression to lytic DNA replication and the extent to which this mechanism mirrors what is observed in α-herpesviruses will be an important step toward advancement of our understating of late gene transcription.

What additional factors are required for true late gene expression? It is now generally accepted that an *Ori*Lyt *in cis* and the βγ gene-encoded vPIC are central to late gene transcription. Given the divergence of late gene transcription from host transcription mechanisms, it is highly probable that additional viral factors are required. Recently, the *BMRF1*-encoded processivity factor has been suggested to play a role in transcription of some late (as well as some latent and early) genes separate from its role in DNA replication [48]. However, the gene array used to measure EBV transcripts in that study [48] is also susceptible to errors due to overlapping transcripts and must be interpreted with caution. Nevertheless, it is notable that BMRF1 (EA-D) is present in quantities vastly exceeding that of the BALF5 catalytic subunit [49], suggesting potential functionality beyond its well-defined role in DNA synthesis [50]. An intriguing possibility is that BMRF1 may serve as a docking site for vPIC–similar to what is seen in the bacteriophage T4 where the gp55/gp33 late gene specific sigma factor links the RNA-polymerase to DNA replication via an interaction with the gp45 processivity factor [51].

The EBV kinase BGLF4 is also implicated in late gene expression [52]. Although BGLF4 has been observed to be localized to the replication compartments [53], its precise role in late gene transcription remains to be defined. Another factor that appears to be important for late gene expression is SM. Specifically, an EBV genome deleted for SM was deficient for expression of a subset of late genes, suggesting that it may play a role in ensuring that their mRNAs are properly exported or otherwise processed as they emerge from replication compartments [54]. Interestingly, disruption of the KSHV SM homolog (ORF57) did not exhibit a late gene defect [55], suggesting that even though SM homologs are present in all herpesviruses, their role in late gene transcription may be unique to EBV.

Finally, a role for Rta in late gene expression has been postulated by many investigators. Rta binding sites are found throughout the EBV genome, potentially allowing activation of nearly any lytic gene promoter [36,44]. Reporter assays; however, offer conflicting evidence regarding their functional significance. Several studies, including our own, have documented RRE-dependent activation of late (or leaky late) promoters [44,56,57]. Aubry et al. have shown that the βγ-encoded vPIC (without presence of Rta) can activate a minimal TATT reporter in EBV-negative HEK293 cells [31]. However, in light of the mounting evidence supporting the central role of replication compartments in both DNA replication and late gene expression [50,58,59], it is unclear the extent to which reporter assays performed in cells not undergoing viral DNA replication (i.e., lacking replication compartments) can be used as models of late gene transcription. Although Rta is undoubtedly essential for late gene expression, what is less clear is whether it plays a direct role or merely supports late gene transcription by fulfilling its essential roles in activation of early gene expression and DNA replication [60]. Experiments to resolve this issue, while extremely important, are difficult to design. If, however, Rta plays only an indirect role in late gene transcription, it would provide an explanation for the role of LF2. Our laboratory has previously shown that LF2 can block EBV replication by sequestration of Rta in the cytoplasm [61,62]. In those experiments, LF2 was transfected simultaneously with Rta. During EBV replication induced by physiologic stimuli, LF2 protein levels would accumulate more slowly and could eventually shutoff Rta-dependent early gene transcription while vPIC dependent late transcription continues. We performed our CAGE-seq analysis using a B95-8 BACmid, a laboratory strain deleted for LF2. Our results suggest that, at least in the absence of LF2, early and late gene transcription compete for limited resources. It is interesting to consider whether, in clinical EBV strains, shutdown of early transcription by LF2 could lead to increased efficiency of late gene transcription once replication compartments have formed under the influence of Rta.

In conclusion, the results reported in this study add to a growing body of evidence that EBV early and late gene transcription are governed by distinct mechanisms. Early gene expression seems to closely resemble that of host gene transcription in which transcription can occur from a chromatinized template. In contrast, we and others have found that late gene transcripts localize to replication factories which are devoid of core histones [30,58,63]. We previously proposed that the major role of the βγ-gene encoded vPIC is to serve as an adaptor complex that exclusively recruits RNA polymerase II to newly replicated viral genomes and facilitates transcription from this atypical unchromatinized template [30]. If such strict requirements are in place for linking late gene transcription to newly replicated templates, how is it then possible for some promoters to exhibit “partial” dependence on DNA replication (i.e., leaky late kinetics)? The results in this study are consistent with the hypothesis that leaky late genes are not transcribed via an entirely distinct mechanism; rather, we propose that they are transcribed by both early (from chromatinized genomes) as well as late (in replication factories) mechanisms. In the case of *BLRF1*, it was possible to distinguish early from late TSS and observe this superimposition directly. A much greater degree of TSS separation occurs with transcription of the CMV UL44 gene [64], where a late TSS is embedded between two early TSS. If the same TSS was used by both early and late mechanisms, each site would appear to be leaky, even if there were no distinct leaky late transcription mechanism.

**Fig 9:**
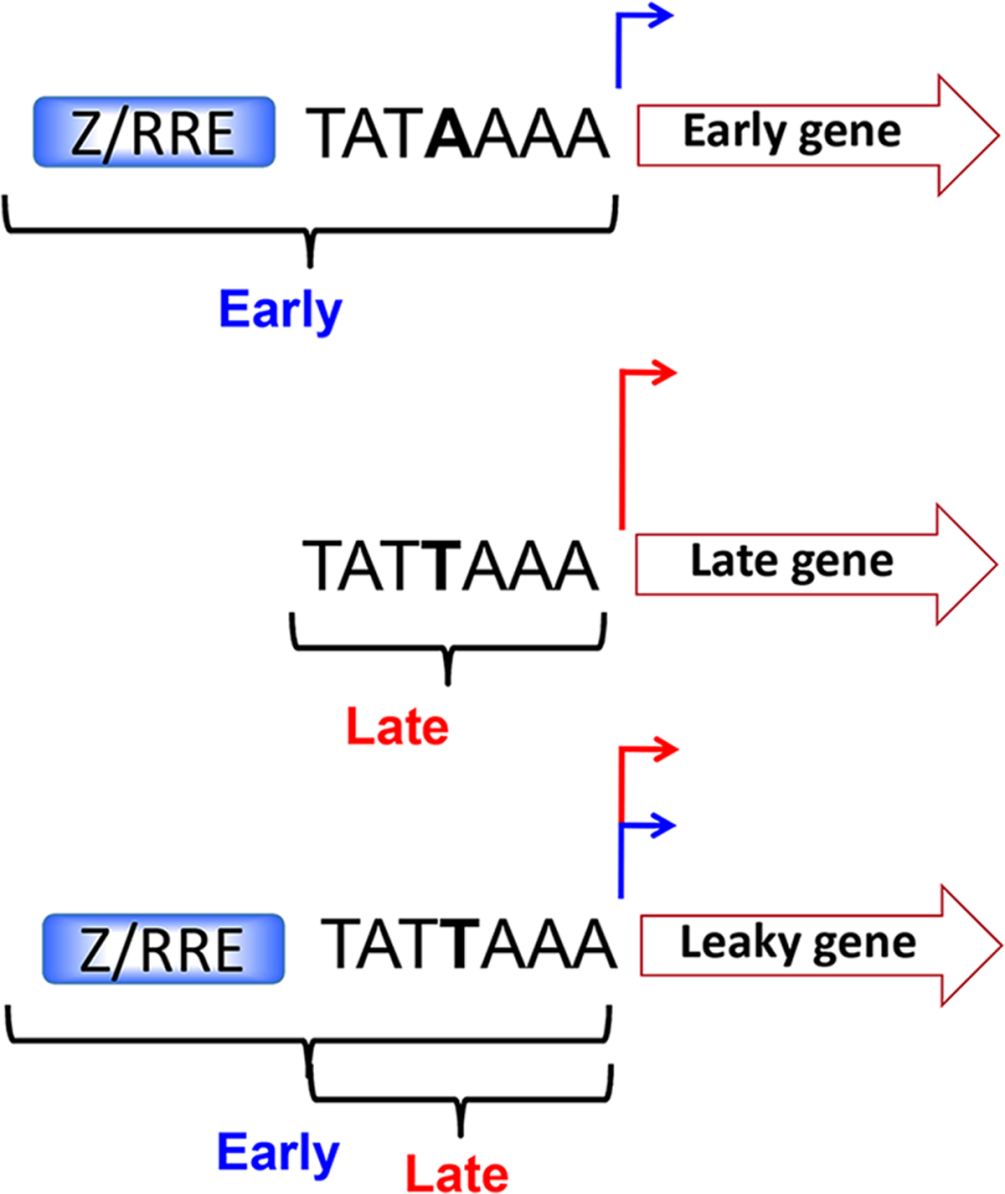
Model for EBV leaky late gene transcription. Current model illustrating early promoters consisting of canonical TATA boxes in addition to upstream Zta and/or Rta response elements (ZREs and/or RREs respectively), true late genes with non-canonical TATT boxes; and hybrid leaky late genes consisting of elements found in both early and late promoters (i.e. ZRE/RRE + TATT).

A corollary of the hypothesis that a leaky late mechanism does not exist *per se* is that leaky late genes have hybrid promoters that support both the early and late transcription mechanisms. There is precedent for this in HSV1, where the VP5 leaky late promoter reverts to early kinetics upon mutation of an initiator element (Inr) known to be important in HSV late promoters [65]. In the case of EBV, we suggest that a leaky late promoter would consist of upstream elements that bind cell transcription factors, an Rta responsive element (RRE) and/or a Zta responsive element (ZRE) with a TATA box capable of binding both hTBP and BcRF1 (vTBP) (model shown in Fig 9). Despite the preference of hTBP and vTBP for TATA boxes with A and T at the fourth position, respectively, they each can bind to the other’s optimal sequence [28,46]. The majority of leaky late gene promoters identified in this study had a T at the fourth position (TATT). It is likely that the presence of strong upstream activators can overcome the reduced affinity of hTBP for TATT, allowing early transcription to also occur at promoters with “late” TATA boxes. Our results demonstrate this directly for the *BLRF2* gene which is transcribed in the absence of BcRF1 (vTBP) with early kinetics, demonstrating the ability of hTBP to bind to its TATT box. In the presence of both DNA replication and BcRF1 (vTBP) leaky late kinetics are observed due to superimposition of early and late transcription. We predict that leaky late transcripts arise from both replication compartments (late) and chromatinized DNA (early) and plan to investigate this hypothesis further using live cell imaging.

## Materials and methods

### Cell lines and culture

All cell lines were maintained in Dulbecco’s modified Eagle medium (DMEM) supplemented with 10% fetal bovine serum (FBS) and 1% penicillin-streptomycin. The EBV-negative HEK293 cell line, used for infection with all EBV BACmids, was obtained from Bill Sugden (University of Wisconsin-Madison).

### EBV mutant genomes and derivation of EBV-positive HEK293 cell lines

The EBV p2089 BACmid contains the complete genome of the B95.8 strain of EBV in addition to a cassette containing the prokaryotic F-factor as well as the green fluorescent protein (*GFP*) and *Hygromycin B* resistance genes in the B95.8 deletion as previously described [66]. The parental EBV Wild-Type (WT) BACmid used in these studies is a modification of the p2089 BACmid lacking a functional *GFP* ORF. The WT, ΔBcRF1 (MI-27), and ΔBDLF4 (MI-84) BACmids were part of a comprehensive library of mutant EBV genomes [67]. ΔBALF2/HA-BcRF1 (referred to as ΔBALF2 elsewhere in this text), and Δ*OriLyt* BACmids have been previously described [30]. ΔBALF2/ΔBcRF1 double mutant was constructed using the GS1783 *E*. *coli*–based *En Passant* method [68,69] by inserting a stop codon in the BALF2 sequence similar to that present in ΔBALF2/HA-BcRF1 [30] in context of the ΔBcRF1 (MI-27) BACmid described above. All EBV-positive HEK293 cell lines were derived as described previously [30].

### Plasmids

pcDNA3-Rta [70], pSG5-Zta (or pSVNaeZ) [71], pMSCV-F-HA-BDLF4 [72], pcDNA3-HA-BcRF1 [30] and pSG5-HA-BALF2 [30] have been previously described.

### Immunoblotting

Total cell lysates were harvested in RIPA Buffer, separated by sodium dodecyl sulfate polyacrylamide gel electrophoresis (SDS-PAGE), and transferred to nitrocellulose membrane. Membranes were blocked in Tris-buffered saline (TBS) containing 5% milk and 0.1% Tween 20 and incubated with appropriate primary antibodies overnight at 4°C. The following primary antibodies were used: anti-EBV EA-D p52/50 (EMD Millipore, MAB8186; 1:3,000), anti-EBV VCAp18 (Thermo Scientific, PA1-73003; 1:1,000), anti-α-tubulin (Sigma, T6074; 1:1,000) and rabbit anti BLRF2 (SLO25-1, generous gift from Ayman El-Guindy; 1:200). Following treatment with primary antibodies, membranes were washed with TBS containing 0.1% tween and incubated with appropriate secondary antibodies for 1 hour at room temperature. The following secondary antibodies were used: goat anti-mouse poly-HRP (Fisher Scientific), goat anti-rabbit poly-HRP (Fisher Scientific), and donkey anti-goat (Fisher Scientific). Membranes were washed again and visualized using ECL chemiluminescent kit (Thermo Scientific) according to manufacturer’s protocol.

### RNA isolation, reverse transcription and quantification by real-time (RT) PCR

EBV-positive HEK293 cells were induced in 12-well plates using 125 ng each of Rta and Zta expression plasmids along with 250 ng of the *trans*-complementing plasmid. 48 hours post lytic induction, cells were washed with phosphate-buffered saline (PBS), and RNA was extracted using GeneJET RNA purification kit (Thermo Scientific) according to manufacturer’s protocol with the following modification: after lysis and before loading on column, lysates was passed through a QIAshredder cell and tissue homogenizer (Qiagen). The eluted RNA was then treated with DNase (1 unit/µg DNA), DNase was deactivated by incubation at 65°C and the treated RNA (~ 1 µg) was reverse transcribed using the ImProm-II Reverse Transcription System (Promega). Purified cDNA was subjected to RT-qPCR with a 7900HT Fast Real-Time PCR system (Applied Biosciences) using SYBR Green Real-Time PCR Master Mix (Biorad). Primers used for detection of β-actin and EBV “BFRF3” (false positive from Fig 1B) are as follows: β-Actin-cDNA-Fwd (GCCGGGACCTGACTGACTAC), β-Actin-cDNA-Rev (TTCTCCTTAATGTCACGCACGAT); FR3-cDNA-qPCR-F (CGGGAGGCTCAAAGAAGTTA), and FR3-cDNA-qPCR-R (GCTCTCTGCCTCTTGTCTATG). All values are reported relative to β-actin mRNA using the 2^-ΔΔ*C*^_T_ method described previously [73].

### non-Amplified non-Tagging Illumina Cap Analysis of Gene Expression (nAnT-iCAGE, referred to as CAGE-seq in text) library preparation and sequencing

Library preparation and sequencing were performed by DNAFORM (Yokohama, Janagawa, Japan) as previously described [34]. Briefly, after RNA assessment by Bioanalyzer (Agilent), first strand cDNAs were transcribed to the 5' end of capped RNAs and attached to CAGE "barcode" tags. Libraries were then sequenced on a NextSeq (Illumina). The raw CAGE-seq data from this study has been deposited in the NCBI SRA under bioproject PRJNA471349.

### Alignment to EBV genome

Reads from CAGE-seq were aligned to the GDC GRCh38 based genome index using STAR [74] version 2.5.1b. Reads were sorted and filtered for primary alignments and mapping quality greater than 30 using samtools version 1.5. Coordinates were converted to Genbank accession V01555.2 coordinates using CrossMap [75].

### Identification of transcription start sites (TSS) clusters and their assignment to annotated ORFs

To identify transcription start sites from CAGE-seq data, reads from bam files containing V01555 coordinates were converted to wigs using a custom python script. A modified version of Paraclu [35] allowing singletons was used to call peaks on the three (pseudo-)wildtypes (WT, BALF2 I + *t*, and BDFL4 I + *t*) with the following parameters: reads per cluster ≥15 and cluster length ≤20. Consensus clusters were identified using a custom python script using Python3, Jupyter Notebook, and Pandas. Briefly, clusters meeting cutoff criteria in at least 2 of the 3 (pseudo-)wildtypes were added to a list of consensus clusters. Partially overlapping clusters were merged. Coordinates of consensus clusters were then applied to calculate the number of tags mapping to each consensus cluster in each sample. Cluster values were normalized to **T**ags **P**er **M**illion (TPM) by dividing the number of reads assigned to each cluster by the total number of mapped reads in each sample (S1 Table). Subsequently, clusters were annotated to known EBV gene products. In cases where more than one cluster was annotated to the same gene, we reported (Table 2) the major cluster (provided all minor clusters were <10% of the major TSS signal) or merged the clusters into a single larger cluster (in cases with multiple TSS lacked a single dominant TSS). Full tracks are available to view in an interactive viewer (https://go.wisc.edu/58sxkb).

### MEME (Multiple EM for Motif Elicitation) analysis of late and leaky late promoters

The MEME software (meme-suite.org; [45]) was used to search for enriched motifs up to 500 bp upstream of late and leaky late TSS (as determined by BALF2 ratios in this study, see Table 2).

## Supporting information

S1 Fig MEME Analysis identifies consensus TATTWAA motif ~ 30 bp upstream of TSS in late and leaky late promoters. MEME analysis showing positions of TATTWAA motif identified in A) late and B) leaky late promoters for 16 late and 15 out 16 leaky late TSS identified via CAGE-seq. BcRF1 is systematically excluded by MEME-suite due to significant deviation from consensus.
(TIF)

S1 Table Kinetics of all EBV lytic transcription start sites (TSS) and their dependence on vPIC as determined by CAGE-seq. Shown are all EBV lytic transcripts identified by CAGE-seq, including strong TSS signals not corresponding to previously annotated ORFs. For each transcription start site (TSS) cluster the table specifies: the location (B95-8, V01555), strand (+ or -), kinetic class based on BALF2 ratio, the gene or promoter corresponding to the observed CAGE-seq TSS cluster, the corresponding annotation for each ORF/promoter, transcript abundance in tags per million (TPM) for each condition, BALF2 ratio (ratio of signal in I / I + *t* for ΔBALF2), and BDLF4 ratio (ratio of signal in I / I + *t* for ΔBDLF4. Clusters that did not correspond to well annotated ORF/promoters are labeled as unknown. Transfection of Rta and Zta resulted in a large number of clusters in this region and, accordingly, have been labeled as artifacts.
(XLS)
