# Supplementary figures and images for: CAGE-seq analysis of Epstein-Barr virus lytic gene transcription: 3 kinetic classes from 2 mechanisms
Source: PLoS Pathog. 2018 Jun 4;14(6):e1007114. doi: 10.1371/journal.ppat.1007114 (PMC6005644; doi:10.1371/journal.ppat.1007114)

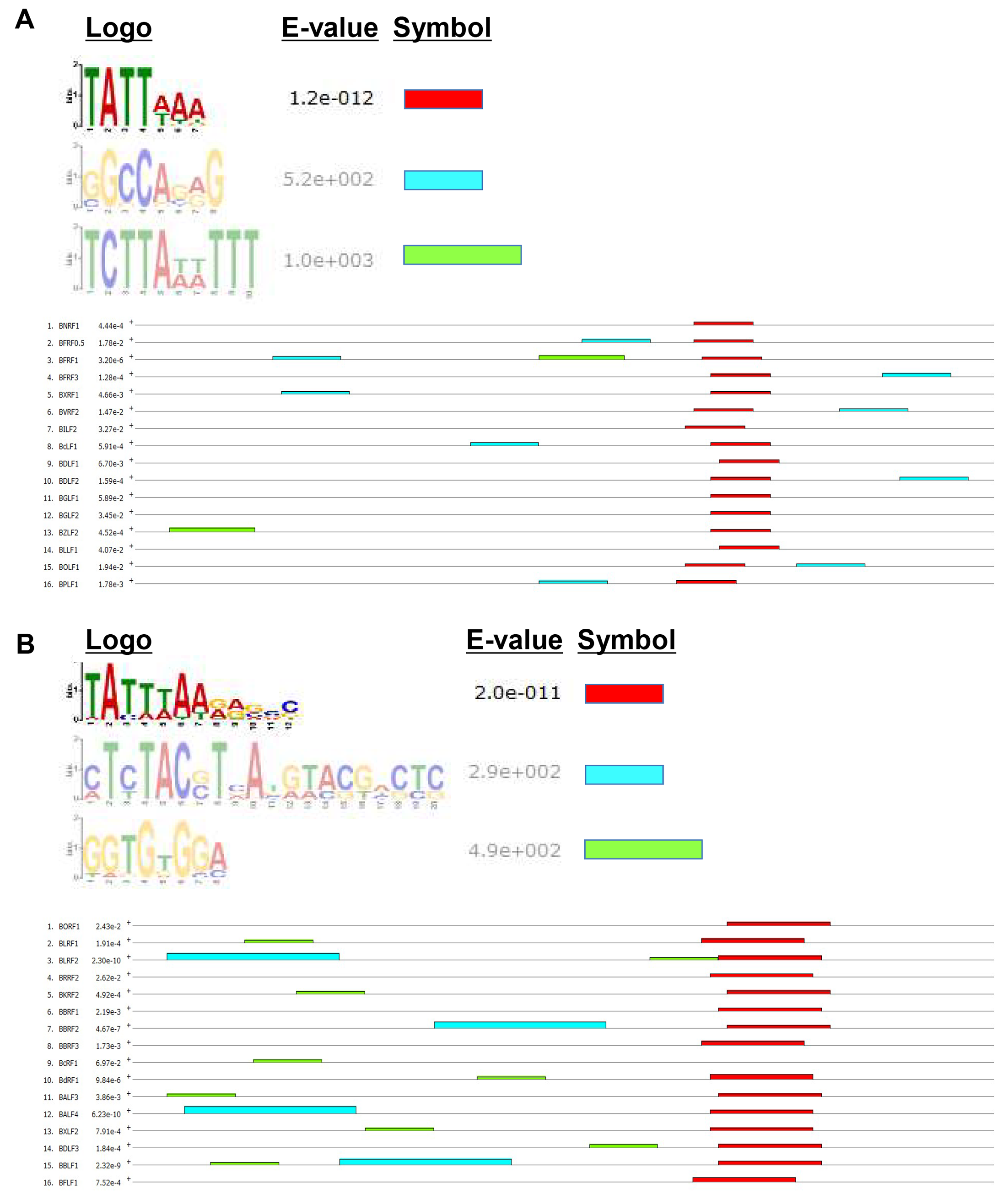

Supplement: S1 Fig — MEME analysis showing positions of TATTWAA motif identified in A) late and B) leaky late promoters for 16 late and 15 out 16 leaky late TSS identified via CAGE-seq. BcRF1 is systematically excluded by MEME-suite due to significant deviation from consensus. (TIF) [file ppat.1007114.s001.tif]
